# Comparison of neoadjuvant chemoradiation with carboplatin/ paclitaxel or cisplatin/ 5-fluoruracil in patients with squamous cell carcinoma of the esophagus

**DOI:** 10.1186/s13014-017-0904-y

**Published:** 2017-11-21

**Authors:** Stefan Münch, Steffi U. Pigorsch, Marcus Feith, Julia Slotta-Huspenina, Wilko Weichert, Helmut Friess, Stephanie E. Combs, Daniel Habermehl

**Affiliations:** 1 Department of Radiation Oncology, Klinikum rechts der Isar, Technical University Munich, Ismaninger Str. 22, D-81675 Munich, Germany; 2 German Cancer Consortium (DKTK), Partner Site Munich, Munich, Germany; 3 Department of Surgery, Klinikum rechts der Isar, Technical University Munich, Ismaninger Str. 22, D-81675 Munich, Germany; 4 Institute of Pathology, Klinikum rechts der Isar, Technical University Munich, Ismaninger Str. 22, D-81675 Munich, Germany; 5 Institute of Innovative Radiotherapy (iRT), Helmholtz Zentrum München, Ingolstädter Landstraße 1, D-85764 Oberschleißheim, Germany

**Keywords:** Squamous cell carcinoma of the esophagus, Neoadjuvant chemoradiation, Cisplatin/5-fluoruracil, Carboplatin/Paclitaxel

## Abstract

**Purpose:**

Neoadjuvant chemoradiation (nCRT) is the treatment of choice for patients with locally advanced squamous cell carcinoma of the esophagus (SCC). Today radiation oncologists can choose between two different therapy regimes including chemoradiation with cisplatin and 5-fluoruracil (CDDP/5FU) and chemoradiation analogue to the CROSS-regime with carboplatin and paclitaxel (Carb/TAX). However, there is a lack of studies comparing these regimes, especially for the subgroup of patients with SCC. In this study, we want to compare nCRT with CDDP/5FU and nCRT with Carb/TAX for patients with locally advanced SCC.

**Patients and methods:**

We retrospectively compared 20 patients who were scheduled for nCRT with a total radiation dose of 41.4 Gy (daily dose of 1.8 Gy) and weekly chemotherapy with carboplatin (Area under the curve 2) and Paclitaxel (50 mg per square meter of body-surface area) according to the CROSS-regime to 31 patients who were scheduled for nCRT with a total radiation dose of 45 Gy (daily dose of 1.8 Gy) and simultaneous chemotherapy with cisplatin (20 mg/m^2^/d) and 5-fluoruracil (500 mg/m^2^/d) on day 1–5 and day 29–33. For the per-protocol (PP) analysis, per protocol treatment was defined as either complete radiation with 41.4 Gy, at least three complete cycles of Carb/TAX and subsequent surgery or complete radiation with 45 Gy, at least one complete cycle of CDDP/5FU and subsequent surgery.

**Results:**

Fifty-one patients (31 patients treated with CDDP/5FU and 20 patients treated with Carb/TAX) were evaluated for the intention-to-treat (ITT) analysis and 44 patients (26 patients treated with CDDP/5FU and 18 patients treated with Carb/TAX) were evaluated for the PP analysis. No significant differences were seen for baseline and tumor characteristics like age, sex, TNM-stage, grading and tumor extension between patients treated with Carb/TAX and patients treated with CDDP/5FU. The most common tumor regression grade after nCRT was grade I as classified by Becker et al., which was observed in 84 and 79% of patients. No significant differences in tumor regression grades were seen between both regimes. Postoperative insufficiency of the anastomosis was seen in 6 patients (33%) who were treated with Carb/TAX and 4 patients (15%) who were treated with CDDP/5FU (*p* = 0.273). Patients treated with CDDP/5FU developed significantly more cumulative hematologic III° (CTCAE) toxicities (58% vs 20%; *p* = 0.010) than patients treated with Carb/TAX. In contrast to that, there was no significant difference for overall survival (OS) and freedom from relapse (FFR) between treatment groups.

**Conclusion:**

In this retrospective analysis, no significant difference was seen for OS and FFR between nCRT with CDDP/5FU and nCRT with Carb/TAX. However, the application of CDDP/5FU was associated with significantly more hematologic III°- toxicities compared to Carb/TAX. Future prospective trials should investigate if these results are reproducible in randomized patient cohorts.

## Introduction

With 400.000 estimated deaths and 456.000 new cases in 2012 esophageal cancer (EC) is the sixth most common cause of cancer death and the eighth most common cancer in the world [1].

In 2008, Tepper et al. [2] demonstrated the superiority of neoadjuvant chemoradiation (nCRT) with cisplatin and 5-fluoruracil (CDDP/5FU) combined with subsequent surgery compared to surgery alone. Multimodal treatment was associated with a significant increase in terms of overall survival and progression-free survival and therefore nCRT with CDDP/5FU and subsequent resection became the treatment of choice for patients with locally advanced squamous cell carcinoma of the esophagus (SCC) suitable for surgery.

However, in the more recent *CROSS*-study, van Hagen et al. [3] compared nCRT with carboplatin and paclitaxel (Carb/TAX) and subsequent surgery to surgery alone in patients with locally advanced EC. Comparable to the results of Tepper et al. patients treated with nCRT with Carb/TAX had a significantly improved overall survival and disease-free survival compared to patients treated with surgery alone. In addition, van Hagen et al. performed a subgroup analysis for patient with SCC and adenocarcinoma (AC) of the esophagus. Thereby, the effect of nCRT was much higher for patients with SCC than for patients with AC.

At out department nCRT with CDDP/5FU was the treatment of choice for locally advanced SCC until 2014. Since then patients with locally advanced SCC were treated analogous to the CROSS-trial. Until now there is only one retrospective analysis comparing nCRT with CDDP/5FU and nCRT with Carb/TAX for patients with EC [4]. In the mentioned study, patients treated with CDDP/5FU had more hematological toxicities, whereas there was no significant difference for overall survival. However, approximately 75% of patients included in this study had AC, while only 24% of patients had SCC. Because the effect of nCRT on overall survival is  higher in patients with SCC compared to patients with AC, there might be a difference in efficiency between nCRT with CDDP/5FU and nCRT with Carb/TAX in SCC patients.

In order to clarify if there should be a preferred chemotherapy regime for SCC patients this study  evaluates and compares toxicity and oncological outcome parameters of these two different nCRT regimes in a homogenous group of SCC patients.

## Patients and methods

Between 2011 and 2016, 51 patients with locally advanced SCC were initially recruited for nCRT with either CDDP/5FU or Carb/TAX at our Department.

### Treatment groups

We retrospectively compared 20 patients who qualified for nCRT with radiotherapy up to a total dose of 41.4 Gy (daily doses of 1.8 Gy) and concomitant, weekly chemotherapy with carboplatin (area under the curve 2) and Paclitaxel (50 mg per square meter of body-surface area) according to the CROSS-regime [3] to 31 patients who were recruited for nCRT with a total radiation dose of 45 Gy (daily dose of 1.8 Gy) and a simultaneous chemotherapy with cisplatin (20 mg/m^2^/d) and 5-fluoruracil (500 mg/m^2^/d) on day 1–5 and 29–33.

For the per-protocol analysis (PP), per protocol treatment was defined as either complete radiation with 41.4 Gy and at least three cycles of Carb/TAX and subsequent surgery or complete radiation with 45 Gy and at least one cycle of CDDP/5FU with subsequent surgery. In summary 7 of 51 patients (14%) were excluded for the per-protocol analysis. One patient of the Carb/TAX group chemotherapy was switched to CDDP/5FU after the first cycle due to an allergic reaction and another one was lost to follow-up before surgery. Within the CDDP/5FU arm one patient was excluded because he received chemotherapy with only cisplatin due to myelodysplastic syndrome and another patient was excluded because the RT protocol was switched to 41.4 Gy due to a high mean lung dose. In one patient therapy was terminated when reaching 27 Gy due to an esophago-tracheal fistula and one additional patient did not undergo surgery due to systemic progression. Another patient was excluded because he did not receive at least 50% of chemotherapy.

### Radiotherapy

All patients in the Carb/TAX group were treated with volumetric modulated arc therapy (VMAT) while 22 patients (71%) in the CDDP/5FU arm were treated with VMAT and 9 patients (29%) were treated with 3-dimensional conformal radiotherapy (3D–CRT). VMAT was done with a median of two arcs [range 1–3] and 3D–CRT was done with a median of 5 [range 5–7] beams.

### Pathology

Resected specimen were available for all 48 patients (94%) who underwent surgery. If no tumor cells reached the margin, the resection was considered as complete and – since in all cases no other metastatic tumor manifestations were present – classified as R0. Response to nCRT was evaluated by extensive and standardized histomorphological workup of resection specimen as described by Becker et al. [5]. In this classification complete tumor regression with 0% residual tumor is classified as grade 1a, subtotal tumor regression with <10% residual tumor per tumor bed is classified as grade 1b, partial tumor regression with 10–50% residual tumor per tumor bed is classified as grade 2 and minimal or no tumor regression with >50% residual tumor per tumor bed is classified as grade 3. WHO grading of tumors (G1, G2, G3) was done on pretherapeutic biopsies.

### Follow- up

Patients were regularly invited to follow-up examinations starting approximately 6 weeks after end of nCRT.

### Toxicity

Acute side effects were retrospectively reviewed using medical records and classified according to the Common Terminology Criteria for Adverse Events (CTCAE) v. 4.03.

### Statistics

In the first step, we performed an intention-to-treat analysis (ITT) with all patients who were scheduled for one of the mentioned therapy regimes. To consider protocol violations we then performed a PP analysis.

Freedom from relapse (FFR) was calculated for patients who underwent surgery. The respective time interval was defined from the day of surgery until tumor progression or tumor recurrence. Overall survival (OS) was defined as the time interval from the beginning of treatment until death. Statistical analyses comprised comparison of baseline parameters, side effects, and different dose parameters using the Wilcoxon–Mann–Whitney U test or Fishers exact test. OS and FFR where compared using the log-rank test. All statistical tests were conducted in an exploratory manner on two-sided 5% significance levels using the software *SPSS Statistics 18 version 18.0.0* (IBM SPSS Statistics, Armonk, U. S.).

## Results

### Baseline and tumor characteristics

Baseline and tumor characteristics for patients included in the ITT and PP analysis are presented in Table 1. Median age of patients was 62 years in both treatment groups and almost two third of patients were male. A total of 75% of all patients in the Carb/TAX group and 87% of all patients in the CDDP/5FU group had uT3 tumors and all except one patient had suspected lymph node metastases in the initial endosonographic staging. Distant metastasis (lung) was seen in one patient of each group. In both of these patients the lung metastases were resected before initiation of nCRT. Median tumor extension was 5 cm (IQR 3–6). All except one patient had moderately (G2, 54%) or poorly differentiated (G3, 44%) tumor grading. Tumor extension was not comprehensible in 2 patients, while grading was not comprehensible in three patients. Median time between end of nCRT and surgery was 38 days [range 9–86 days]. In summary, no significant differences in baseline parameters were seen between patients treated with Carb/TAX and patients treated with CDDP/5FU.

**Table 1:** Patients’ and tumor characteristics

Parameter	Intention to treat			Per protocol		
	*Carb/TAX n* = 20	CDDP/5FU *n* = 31	*p*-value	*Carb/TAX n* = 18	CDDPs/5FU *n* = 26	p-value
Median Age (IQR)	62 (56–71)	62 (55–72)	0.875	61 (56–69)	65 (57–72)	0.489
Male	13 (65%)	20 (65%)	1.000	13 (72%)	16 (62%)	0.531
T-stage			0.143			0.103
uT1	0 (0%)	1 (3%)		0 (0%)	1 (4%)	
uT2	5 (25%)	2 (6%)		5 (28%)	2 (8%)	
uT3	15 (75%)	27 (87%)		13 (72%)	23 (88%)	
uT4	0 (0%)	1 (3%)		0 (0%)	0 (0%)	
uN+	20 (100%)	30 (97%)	1.000	18 (100%)	25 (96%)	1.000
cM0	20 (100%)	31 (100%)	1.000	18 (100%)	26 (100%)	1.000
Grading			1.000			0.765
G1	0 (0%)	1 (3%)		0 (0%)	0 (0%)	
G2	11 (58%)	15 (52%)		11 (61%)	14 (56%)	
G3	8 (42%)	13 (45%)		7 (39%)	11 (44%)	
Tumor extension (IQR)	4 (2–6)	5 (3–7)	0.233	4 (2–6)	5 (3–6)	0.232

*5-FU* 5-fluoruracil, *IQR* inter-quartiles-range

### Pathology

Resection status was insecure (RX) in three patients (6%), due to tissue artifacts induced by complex surgical procedures in complicated cases. However, while complete resection (R0) was achieved in 44 of the remaining 45 patients (98%), in one patient (2%) treated with Carb/TAX resection status was classified as R1. No significant difference was seen for the distribution of tumor regression grade after nCRT (Table 2). The most common tumor regression grade was grade I (< 10% residual tumor) which was seen in 84% of patients (ITT and PP) treated with the CROSS regime and 79% (ITT) and 80% (PP) of patients treated with CDDP/5FU.

**Table 2:** Tumor regression grade

Tumor regression	Intention to treat			Per protocol		
	*Carb/TAX n* = 19	*CDDP/ 5FU n* = 29	*p*-value	*Carb/TAX* n = 18	*CDDP/ 5FU* n = 26	*p*-value
Ia (pCR)	5 (26%)	11 (38%)	0.731	5 (28%)	10 (38%)	0.721
Ib	11 (58%)	12 (41%)		10 (56%)	11 (42%)	
2	1 (5%)	3 (10%)		1 (6%)	3 (12%)	
3	2 (11%)	3 (10%)		2 (11%)	2 (8%)	

*pCR* pathologic complete remission

### Toxicities

In one patient of the CDDP/5FU group therapy was terminated when reaching 27 Gy due to an esophago-tracheal fistula. No significant differences were seen for the proportion of patients suffering from postoperative insufficiency of the anastomosis. Referring to the per-protocol population 6 patients (33%) who were treated with Carb/TAX and 4 patients (15%) who were treated with CDDP/5FU suffered from insufficiency of the anastomosis (*p* = 0.273).

Regarding hematologic parameters significantly more grade III toxicities were seen in patients treated with CDDP/5FU (58%) compared to patients treated analogue to the CROSS-protocol (20%) (*p* = 0.010). Within the intention-to-treat population a leukopenia I°, II°, III° and IV° was observed in 20, 50, 20 and 0% of patients treated with Carb/TAX, respectively. In contrast to that, leukopenia grade I°, II°, III° and IV° was seen in 13, 19, 48 and 10% of patients treated with CDDP/5FU (*p* = 0.065). In patients treated per protocol, significantly higher rates of leukopenia were seen in patients who received CDDP/5FU than in patients who were treated with Carb/TAX (I°: 15% vs. 17%, II°: 19% vs. 56%, III°: 50% vs. 17%, IV°: 8% vs. 0%; *p* = 0.048).

No significant differences were seen for thrombocytopenia (*p* = 0.654) and anemia (*p* = 0.364) between treatment groups. All hematologic side effects are shown in Table 3.

**Table 3:** Hematologic toxicities

Hematologic toxicities	Intention to treat			Per protocol		
	*Carb/TAX* *n* = 20	*CDDP/5FU* *n* = 31	*p*-value	*Carb/TAX* *n* = 18	*CDDP/5FU* *n* = 26	*p*-value
≥ III°	4 (20%)	18 (58%)	**0.010**	3 (17%)	15 (58%)	**0.012**
Leukopenia			0.065			**0.048**
0°	2 (10%)	3 (10%)		2 (11%)	2 (8%)	
I°	4 (20%)	4 (13%)		3 (17%)	4 (15%)	
II°	10 (50%)	6 (19%)		10 (56%)	5 (19%)	
III°	4 (20%)	15 (48%)		3 (17%)	13 (50%)	
IV°	0 (0%)	3 (10%)		0 (0%)	2 (8%)	
Thrombocytopenia			0.654			0.364
0°	15 (75%)	18 (58%)		14 (78%)	14 (54%)	
I°	2 (10%)	6 (19%)		2 (11%)	6 (23%)	
II°	2 (10%)	2 (6%)		2 (11%)	2 (8%)	
III°	1 (5%)	4 (13%)		0 (0%)	3 (12%)	
IV°	0 (0%)	1 (3%)		0 (0%)	1 (4%)	
Anemia			0.184			0.479
0°	3 (15%)	0 (0%)		2 (11%)	0 (0%)	
I°	10 (50%)	19 (61%)		10 (56%)	16 (62%)	
II°	6 (30%)	9 (29%)		5 (28%)	7 (27%)	
III°	1 (5%)	3 (10%)		1 (6%)	3 (12%)	

Bold numbers indicate statistical significance with *p* <0.05

### In-hospital-mortality

Two patients (12%) in the Carb/TAX group and 3 patients (12%) in the CDDP/5FU group died before leaving the hospital after surgery (*p* = 1.000). Median time from surgery to death in these patients was 0.97 months. In three of five patients who died during hospitalization, death was caused by postsurgical acute respiratory distress syndrome without any signs for insufficiency of the anastomosis. One patient died from pulmonary complications due to an insufficiency of the anastomosis. The last patient died from sepsis with multiorgan failure, probably caused by an insufficiency of the anastomosis.

### Treatment failure

In summary, local or distant treatment failure was seen in 4 (22%) patients (Carb/TAX) and 8 (31%) patients (CDDP/5FU) of the per-protocol population (*p* = 0.723) and in 4 (21%) patients patients (Carb/TAX) and 10 (34%) patients (CDDP/5FU) of the ITT-population (*p* = 0.354).

Loco-regional recurrence was the most common reason for treatment failure. Within the per-protocol population, loco-regional failure and distant failure was seen in 3 patients (17%) and 2 patients (11%) treated with Carb/TAXc, respectively. Compared to that, loco-regional failure and distant failure was seen in 6 patients (23%) (*p* = 0.716) and 3 patients (12%) (*p* = 1.000) treated with CDDP/5FU.

### Survival

**Fig. 1:**
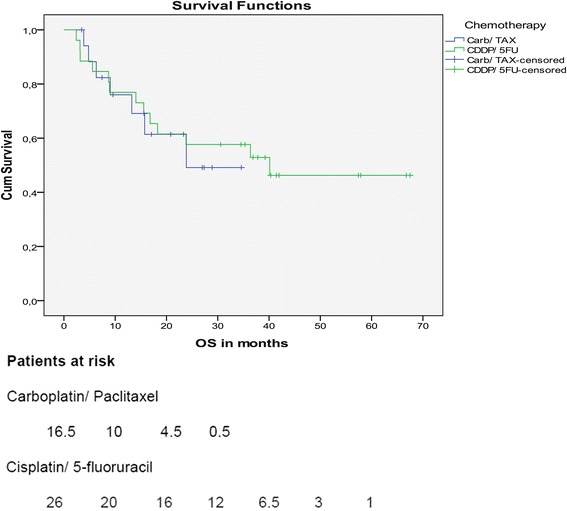
Overall survival. Patients at risk: Carboplatin/ Paclitaxel - 16.5, 10, 4.5, 0.5. Cisplatin/ 5-fluoruracil - 26, 20, 16, 12, 6.5, 3, 1.

After a median follow-up of 34.6 months for surviving patients in the per-protocol population, median overall survival (OS) was 23.9 months for patients treated with Carb/TAX and 40.1 months for patients treated with CDDP/5FU. This difference was not statistically significant (*p* = 0.802) (Fig. 1).

**Fig. 2:**
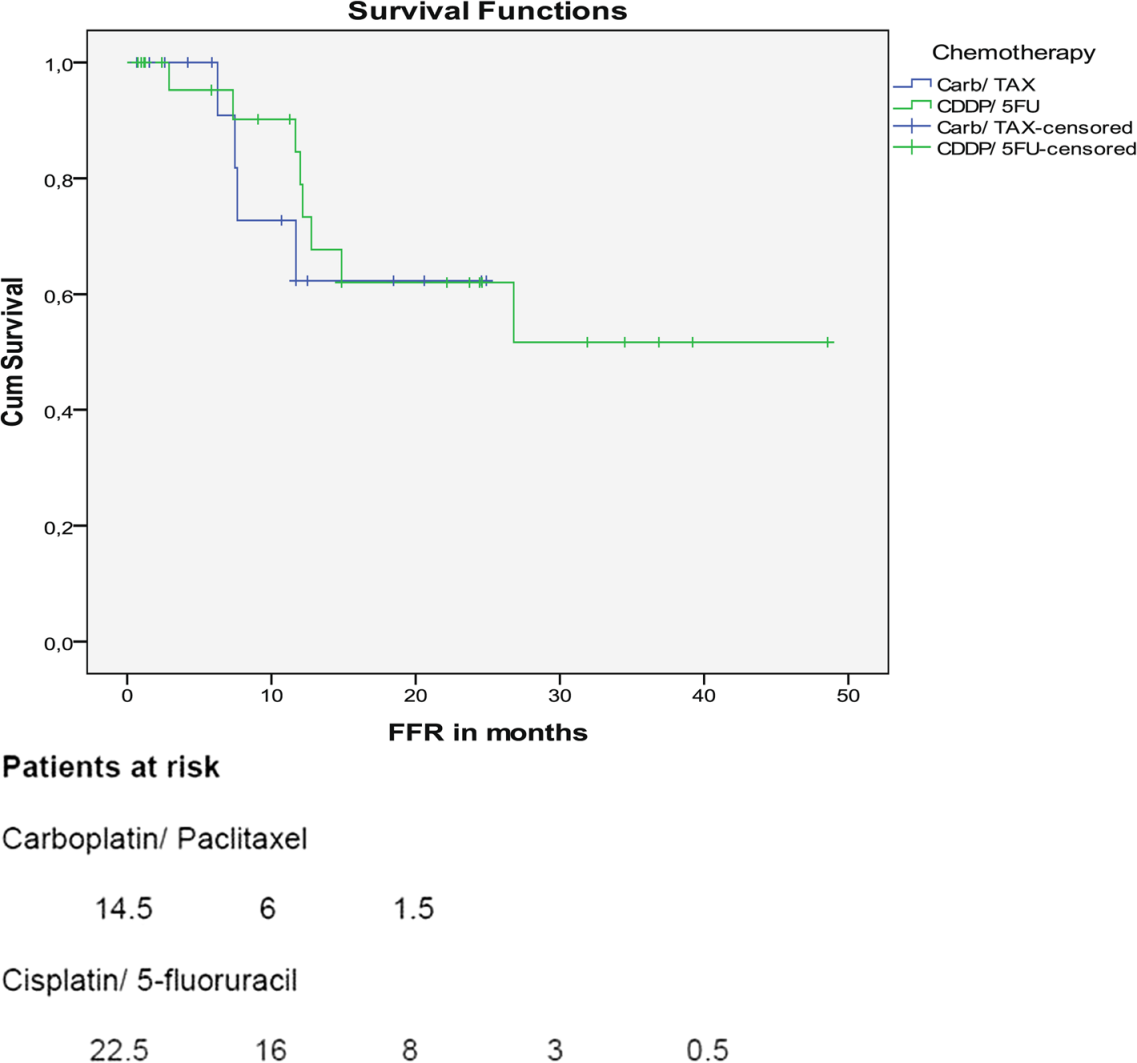
Freedom from relapse. Patients at risk: Carboplatin/ Paclitaxel - 14.5, 6,1.5. Cisplatin/ 5-fluoruracil - 22.5, 16, 8, 3, 0.5.

Median FFR was not reached in both patient groups. However, no significant difference was seen for patients treated with Carb/TAX or CDDP/5FU (*p* = 0.696) (Fig. 2).

## Discussion

nCRT is the treatment of choice for patients with locally advanced SCC of the esophagus, suitable for surgery [6, 7]. Although different treatment protocols for nCRT have successfully improved overall survival and complete resection rates compared to surgery alone in EC patients, head-to-head comparisons with a competitive focus on oncologic efficacy and toxicity are still missing. The most widely used concomitant chemotherapy protocols are currently based on CDDP/5FU or Carb/TAX. In this analysis, we retrospectively compared nCRT according to the CROSS-protocol to nCRT with CDDP/5FU in a slightly modified version of the regime described by Bates et al. [8].

While there were no differences regarding overall survival and freedom from relapse for the two treatment regimes, significantly more hematologic III° toxicities were observed in patients treated with CDDP/5FU. These findings are consistent with the results of Blom et al. [4]. In their study, the authors compared nCRT analogue to the CROSS-regime with nCRT analogue to the regime published by Tepper et al. [2] for 165 patients with SCC or adenocarcinoma of the esophagus. No significant differences were seen for OS, but as in our study, patients treated with CDDP/5FU had a significantly higher risk of developing any grade III toxicity than patients treated with the CROSS-regime (41% vs. 25%). Thereby, the most common ≥ III° toxicity was leukopenia, which was observed in 24% of patients treated with CDDP/5FU and 10% of patients treated with the CROSS-regime. In our study we also recognized a significant difference regarding the incidence of ≥ III° hematologic toxicities (58% vs. 20%, ITT and 58% vs. 17%, PP). Especially for patients treated with Carb/TAX the rate of hematologic ≥ III° toxicity in general as well as the rate of anemia, leukopenia and thrombocytopenia in particular was much higher than in other studies [3, 4, 9]. Nonetheless, toxicity results for patients treated with CDDP/5FU match with the results by Tepper et al. [2] (57% vs. 58% of patients with hematologic ≥ III° toxicity) while two other studies report significant less hematologic toxicity in patients treated with cisplatin and 5-fluoruracil (15–17%) [4, 8]. However, because the difference in radiation dose between treatment groups was just 3.6 Gy our results underlines that the difference in hematologic toxicity is probably caused by the different chemotherapy regime. As in other studies leukopenia was the most common hematologic toxicity and our analysis showed a strong trend for higher grades of leukopenia in patients treated with CDDP/5FU in the ITT-analysis and significantly higher grades of leukopenia in patients treated with CDDP/5FU in the PP population.

Regarding non-hematologic side effects no significant difference was seen for the rate of insufficiencies of the anastomosis and the rate of in-hospital-mortality between both treatment regimes. However, the absolute difference in the rate of insufficiencies of the anastomosis is remarkable. While it was seen in 15% of patients who received CDDP/5FU, which is comparable to the results by Blom et al. [4] (13% vs. 15%), 33% of patients treated with Carb/TAX were diagnosed with postsurgical insufficiency of the anastomosis. These results should be kept in mind and reviewed in further trials, especially because we don’t have a convincing explanation.

In accordance with other studies no significant difference was seen for OS and FFR between both treatment groups [4, 9]. However, for patients treated with Carb/TAX median OS was significant shorter than in the CROSS-trial [3, 10]. In the CROSS-trial median OS of patients with SCC was 81.6 months. In a retrospective trial the median OS for EC patients treated with Carb/TAX was not reached after 3 years [4]. Because OS after trimodal therapy is better for patients with SCC than for patients with AC, we have to point out that in this trial only 24% of patients had SCC. For patients treated with CDDP/5FU median overall survival is comparable to other published results (17.7–53 months) [2, 4, 8, 11]. The short median OS of 17.7 months within the FFCD 9102 trial [11] (89% SCC) is probably associated with the high rate of local treatment failure (34%). Radiotherapy in this trial was done with either a conventional regime (46 Gy in 23 fractions) or as a split-course regime with 5 fractions and a daily dose of 3 Gy in the first and fourth week of treatment. In summary, the reason for the reduced median OS in patients treated according to the CROSS protocol remains unclear. However, when looking to the rate of local or distal disease progression our results show no increased incidence of treatment failure compared to the long-term data of the CROSS Trial [10]. Results for alternative chemotherapy regimens were reported in two further trials for patients undergoing definite chemoradiation for EC [12, 13]. In a phase II trial by Wolf et al. [12] 135 patients (85% SCC) received dCRT with mitomycin C and 5-fluoruracil. Radiation dose was more than 54 Gy in 85% of patients. Xia et al. [13] evaluated dCRT with weekly paclitaxel and 5-fluoruracil and simultaneous radiotherapy (50.4 Gy or 61.2 Gy) for 53 patients (94% SCC). Median OS was 15.6 months and 17.9 months, respectively. However, comparability is restricted, because patients were treated with dCRT instead of nCRT with subsequent surgery. While no further information about toxicity were given by Wolf et al. ≥ III° neutropenia was seen in 7% of patients treated with paclitaxel and 5-fluoruracil [13].

In our study complete tumor regression (pCR, Becker Ia) was seen in 28% (Carb/TAX) and 38% (CDDP/5FU) of patients, respectively. These results match with the results of the retrospective trial published by Blom et al. and van Meerten et al. [4, 14]. While these studies included patients with SCC and AC (22–24% SCC) studies focusing on only SCC patients report higher rates of pCR after nCRT with CDDP/5FU (51%) or Carb/TAX (49%) [3, 8]. This might be partially explained by the diagnostic tissue workup, because in our center we pursue a very rigorous course and pCR can only be stated when the whole tumor area has been subjected to histological workup. In accordance to the results of Blom et al., no statistically significant difference regarding the proportion of tumor regression grades was seen between both nCRT regimes [4]. An overview of different studies evaluating pCR, overall survival and hematologic toxicity of different nCRT regimes for SCC is given in Table 4.

**Table 4:** Overview of studies evaluating different nCRT regimes for EC

Author (year)	*N*° of patients	Study design	Histology	RTx	CTx regime	Median OS in months	Local failure rate	pCR rate	Hemato-logic toxicity (≥ III°)
Bates [8] (1996)	35	Phase 2	80% SCC	45 Gy (25 Fx)	Cisplatin 5FU	25.8	7%	51%	17%
Tepper [2] (2008)	30 (trimodal therapy)	Phase 3	23% SCC	50.4 Gy (28 Fx)	Cisplatin 5FU	53.8	13%	40%	57%
Bosset [18] (1997)	143 (trimodal therapy)	Phase 3	100% SCC	18.5 Gy (5 Fx)	Cisplatin (0–2 days before first day of radiotherapy)	18.6	–	26%	–
van Hagen; Shapiro [3, 10] (2012; 2015)	178 (trimodal therapy)	Phase 3	23% SCC	41.4 Gy (23 Fx)	Carboplatin Paclitaxel	48.6 (all) 81.6 (SCC)	22%	29% (all) 49% (SCC)	8%
Bedenne [11] (2007)	129 (trimodal therapy)	Phase 3	89% SCC	46 Gy (23 Fx) or 15 Gy (5 Fx) – week 1 + 4	Cisplatin 5-Fluoruracil	17.7	33.6%	–	–
Urba [19] (2003)	69	Phase 2	14% SCC	45 Gy (30 Fx) – twice daily	Cisplatin Paclitaxel	24	–	22%	13%^a^
Blom [4] (2014)	165	Retro-spective analysis	24% SCC	1. 50.4 Gy (28 Fx) 2. 41.4 Gy (23 Fx)	1. Cisplatin 5-Fluoruracil 2. Carboplatin Paclitaxel	Not reached after 3 years	–	38% 24%	1.15%^a^ 2.2% ^a^
van Meerten [14] (2006)	54	Phase 2	22% SCC	41.4 Gy (23 Fx)	Carboplatin Pacliataxel	Not reached after median follow up of 31 months	13%	25%	25% ^a^
This analysis	51	Retro-spective analysis	100% SCC	1. 45 Gy (25 Fx) 2. 41.4 Gy (23 Fx)	1. Cisplatin 5-Fluoruracil 2. Carboplatin Paclitaxel	1. 40.1 2. 23.9	1. 34% 2. 21%	1. 38% 2. 26%	1. 58% 2. 20%

^a^ neutropenia, *RTx* radiotherapy, *CTx* chemotherapy, *SCC* squamous cell carcinoma, *Fx* fractions

In our study, all patients (100%) undergoing nCRT with Carb/TAX and 71% of patients treated with CDDP/5FU received radiotherapy using VMAT. This is important, because different trials have already demonstrated the superiority of VMAT over 3D–CRT for radiotherapy of esophago-gastric cancer regarding dose reduction to the organs at risk when compared to 3D–CRT [15–17]. However, it remains questionable if these dosimetric differences alone can lead to higher rates of hematological side effects. In an analysis comparing VMAT and 3D–CRT for nCRT of esophageal cancer the use of VMAT was associated with higher rates of leukopenia when compared to 3D–CRT [16], but we have to point out that chemotherapy regime was not standardized. Therefore, the used chemotherapy regime seems to be more important for acute hematologic side effects.

Due to its retrospective nature this study has some limitations. The first problem is the relatively small number of patients in each treatment group. However, to the best of our knowledge this study includes the first dataset comparing nCRT with CDDP/5FU or Carb/TAX for patients with only SCC. Another problem is the imbalance concerning follow-up time. Because nCRT with CDDP/5FU was only used until 2014 and nCRT with Carb/TAX was only used since then, follow-up for patients treated with CDDP/5FU is obviously longer than for patient treated with Carb/PTAX, which can affect OS and FFR. Due to these limitations, the significance of the results is clearly impaired. So when looking to the fact that we could find no significant differences in terms of survival and treatment response between both groups, we have to keep in mind that the relatively small number of patients limits the power of the study.

## Conclusion

In this retrospective analysis, no significant difference was seen for OS and FFR between nCRT with CDDP/5FU and nCRT with Carb/TAX. However, the application of CDDP/5FU was associated with significantly more hematologic III°- toxicities compared to Carb/TAX. Future prospective trials should investigate if these results are reproducible in randomized patient cohorts.

## Abbreviations

3D–CRT: 3-dimensional conformal radiotherapy
AC: Adenocarcinoma
Carb/TAX: Carboplatin and Paclitaxel
CDDP/5FU: Cisplatin and 5-fluoruracil
CTCAE: Common terminology criteria for adverse events
EC: Esophageal cancer
FFR: Freedom from relapse
Gy: Gray
ITT: Intention-to-treat
nCRT: neoadjuvant chemoradiation
OS: Overall survival
pCR: Pathologic complete response
PP: Per-protocol
SCC: Squamous cell carcinoma
VMAT: Volumetric modulated radiotherapy
